# Intranasal oxytocin treatment on the day of weaning does not decrease walking behavior or improve plasma metabolites in beef calves placed on pasture

**DOI:** 10.1093/tas/txab191

**Published:** 2021-10-02

**Authors:** Kirsten R Nickles, Alejandro E Relling, Anthony J Parker

**Affiliations:** Department of Animal Sciences, The Ohio State University, Wooster, OH 44691, USA

**Keywords:** behavior, global positioning system, stress response, welfare

## Abstract

The purpose of this study was to evaluate the effect of intranasal oxytocin administered at abrupt weaning on weaning behaviors such as walking distance and time devoted to walking, calf body weight, and plasma non-esterified fatty acids (NEFAs), β-hydroxybutyrate (β-HB), and cortisol. Twenty Simmental × Angus heifer calves were randomly assigned to one of two treatments: intranasal oxytocin (OXT; *n* = 10) or intranasal saline (CON; *n* = 10). All calves were given the respective intranasal treatment on the day of weaning (day 0) and then placed on pasture together. Calves were weighed and a blood sample was obtained on days 0, 1, 7, and 14 postweaning. Blood samples were subsequently used to quantify plasma NEFA, β-HB, and cortisol concentrations. All calves in both treatment groups were fitted with an individual global positioning system that recorded calf location every 10 s for a 16-h period on days 0, 7, and 14 to quantify and evaluate walking behaviors. There was no treatment × day effect for distance walked (*P* = 0.82), walking time (*P* = 0.80), non-walking time (*P* = 0.88), area utilization index (*P* = 0.84), calf body weight (*P* = 0.82), average daily gain (*P* = 0.54), NEFA (*P* = 0.22), or cortisol concentrations (*P* = 0.32). There was a tendency for a treatment × day effect observed for average walking speed (*P* = 0.09) and β-HB (*P* = 0.10), such that calves in the CON treatment tended to have lesser average walking speeds on day 14 and tended to have greater β-HB concentration after weaning. There was a treatment effect (*P* = 0.02) for NEFA concentrations, with the CON calves having a greater plasma NEFA concentration throughout the study compared with OXT calves. These data imply that OXT calves may have had differing metabolic responses immediately after weaning that could have altered the mobilization of NEFA, but this change was not substantial enough to impact body weights or walking behaviors.

## INTRODUCTION

Weaning beef calves prepares the calf to enter the next phase of growth and development and decreases the nutritional and production requirements of the cow (Enríquez et al., 2011). The weaning process can be stressful because it may subject a calf to several husbandry and management practices at the same time such as frequent handling, vaccination, castration, transportation, mixing with unfamiliar cattle, movement to a new environment, and a change in diet (Lynch et al., 2019). The accumulation of experiences at weaning can have short- and longer-term effects on the physiological and psychological status of the calf. For example, beef calves engage in an abnormal time spent walking and distance walked at weaning; however, abnormal walking behavior is reported to cease within 7 d after weaning (Nickles et al., 2020). Weaning decreases dry matter intake of the calf and this decreases body weight growth for up to 21 d after weaning (Gibb et al., 2000; Loerch and Fluharty, 2000; Haley et al., 2005; Weary et al., 2008; Campistol, 2010). Plasma cortisol concentration is often used as a key physiological indicator of an animal’s response to novel experiences. Abrupt weaning has been reported to increase plasma cortisol concentration when compared with the plasma cortisol concentration of unweaned calves (Hickey et al., 2003). Increased plasma cortisol concentration has been well documented in ruminants subjected to novel experiences; however, other hormones, such as oxytocin, have been reported to have an association with plasma cortisol concentration (Yayou et al., 2008; Wagner et al., 2020b). Oxytocin has been hypothesized to be potentially involved in the attenuation of the hypothalamic–pituitary–adrenal (HPA) axis (Tilbrook and Clarke, 2006). In lactating sheep, a greater plasma oxytocin concentration was associated with an attenuated response in plasma cortisol when ewes were confronted with a barking dog (Ralph and Tillbrook, 2016). Furthermore, intracerebroventricularly infused oxytocin decreased the plasma cortisol concentration in Holstein steers (Yayou et al., 2008). Intranasal oxytocin treatment, however, failed to attenuate the adrenocorticotropic hormone or cortisol response to restraint and isolation stress in beef heifers. The relationship between plasma oxytocin and cortisol concentration appears to be dependent upon the species, the stressor used, previous experience of the animal, the genetic type of the animal, and the environment (Wagner et al., 2020a). Wagner et al. (2020b) speculated that exogenous oxytocin treatment may attenuate the HPA axis at less fearful experiences than the restraint and isolation model that they utilized with beef heifers. Intranasal oxytocin treatment may be a novel strategy that beef producers can use to minimize the response of the calf’s HPA axis to the weaning process. We hypothesized that treating calves with intranasal oxytocin at weaning would attenuate plasma cortisol concentration and decrease the distance and time spent walking at weaning. We further hypothesized that less walking by the calves would have a positive effect on calf body weight and metabolic status observed through decreased plasma nonesterified fatty acid (NEFA) and β-hydroxybutyrate (β-HB) concentration. Based on these hypotheses, the objective of the current study was to assess the impact of intranasally administered oxytocin on calf growth and metabolic responses during the postweaning period.

## MATERIALS AND METHODS

All procedures were approved by The Ohio State University Institutional Animal Care and Use Committee (Animal Use Protocol #2018A00000085). The following study was completed in September 2018 at the Jackson Agricultural Research Station (JARS; Jackson, OH, USA).

### Animals and Management

A total of 20 Angus × Simmental heifer calves (162 ± 9 d of age; 194 ± 7.4 kg body weight (BW)) were used in this weaning study. All calves were born and remained at JARS for the duration of the study. Calves were not creep fed prior to weaning and were only allowed to nurse and consume pasture forage. At weaning, calves were vaccinated with a modified live virus (Bovi-Shield Gold 5, Zoetis Services LLC., Parsippany, NJ, USA) against infectious bovine rhinotracheitis, bovine viral diarrhea (types 1 and 2), parainfluenza 3, and bovine respiratory syncitial virus viruses, and a bacterin-toxoid for the prevention of bovine pneumonia (One Shot, Zoetis Services LLC., Parsippany, NJ, USA). All 20 calves were also de-wormed (Dectomax Pour-On, Zoetis Services LLC., Parsippany, NJ, USA) at the time of vaccination.

On the day of weaning (day 0), the 20 heifer calves were randomly allocated to one of two treatment groups: intranasal saline (CON; *n* = 10) or intranasal oxytocin (OXT; oxytocin 0.66 IU/kg BW; *n* = 10). Treatments were only given on the day of weaning (day 0). Dose rates for the saline treatment were applied in the same manner as the oxytocin to keep dosing amounts similar for both treatments (Isotonic saline 0.9% or Oxytocin, 20 IU/mL Vetone, Bimeda-MTC Animal Health Inc., ON, Canada). The dose rate used in this study was chosen based on a pharmacokinetic study described by Wagner et al. (2020a) in which doses of 0.33, 0.66, and 1.32 IU oxytocin/kg BW were administered to beef steers by the intranasal route and resulted in similar maximum concentration, time of maximum concentration, area under the curve extrapolated to infinity, clearance by fraction dose, half-life, and mean residence time. Thus, the middle dose rate was chosen. Doses ranged from 5.5 to 7 mL, dependent upon individual calf body weight. Half of each dose was administered into each nostril with a mucosal atomization device (Nasal Teleflex Inc., Morrisville, NC). After receiving their treatments, all calves were housed together in the same pasture and were provided 0.91 kg/animal/d of a 17% protein starter diet (Table 1). Calves were provided supplement each day and consumed all of the supplement that was provided. Due to weather and forage conditions, calves were moved when necessary to a new pasture to allow for adequate grazing. From days 0 to 6, calves were placed in a 1-hectare pasture; on days 7 to 9, calves were moved to a 0.4-hectare pasture; and on days 10 to 15, calves were moved to a 1.3-hectare pasture. Forage was available for calves to graze and was estimated to be between 2,100 and 2,500 kg dry matter (DM)/ha at the start of the treatment period and was in a vegetative state when calves were moved to a new pasture. 

**Table 1.** Calf starter supplement composition

Ingredient	% DM basis
Ground corn	52.79
Soybean meal	3.05
Low-fat dried distiller’s grain	18.01
Corn gluten feed	20.64
Limestone	1.15
Trace mineral salt	0.44
Vitamin A	0.06
Vitamin D	0.06
Vitamin E	0.02
Selenium	0.03
*Aspergillus oryzae* fermentation product (141 g/kg concentrate)	0.77
Blended animal & vegetable fat	2.98

### Global Positioning System Collars

All calves in each treatment (OXT, CON) had a collar placed around their neck with a Garmin Astro 430/T5 GPS/GLONASS global positioning system (GPS) tracking device (Garmin International, Inc., Olathe, KS) at weaning on day 0 at approximately 0800 h when calves were simultaneously weighed and sampled for blood. Each collar remained on the calves for a 24-h period and was then removed to perform battery recharging. The GPS devices were calibrated to receive a GPS position of the calves every 10 s, and all collars recorded from 2300 h on days 0, 7, and 14 to 1500 h on days 1, 8, and 15 after weaning to provide for a common 16 total hours of tracking at these three time points for all calves without any missing data points. The data extracted from the collars were total distance traveled (km; total distance walked during the 16-h period), walking time (h; total time spent walking during the 16-h period), non-walking time (h; total time spent on all other activities besides walking during the 16-h period), area utilization index (AUI; calculated by determining the polygonal area that each calf walked during 16 h and dividing this area by the total area of the pasture provided), and average speed (m/s; average walking speed during the 16-h period). These data were obtained using the Garmin BaseCamp software (Garmin International, Inc., Olathe, KS). All of these variables were standard output from the Garmin BaseCamp software; however, AUI was created using the Garmin BaseCamp standard area output. All data were recorded for each individual heifer (*n* = 20).

The GPS collars were reported by Garmin to be accurate to within 15 m 95% of the time when there were no physical obstructions of the system to the celestial sphere (https://www.garmin.com/en-US/).

### Temperature–Humidity Index

Weather data including minimum, maximum, and average ambient air temperature and relative humidity were collected daily using the JARS weather station (https://weather.cfaes.osu.edu/stationinfo.asp?id=5). From these data, a minimum, maximum, and average temperature–humidity index (THI) was calculated for each day of the study using the following equation from Mader et al. (2006): THI = 0.8 × ambient temperature + [(% relative humidity ÷ 100) × (ambient temperature – 14.4)] + 46.4.

### Sampling and Analysis

Heifer calves were abruptly weaned from their dams on day 0 when they received their intranasal treatment. All calves were sampled for blood (10 mL) via jugular venipuncture into a vacutainer tube containing lithium heparin and body weight on days 0, 1, 7, and 14 at approximately 0800 h. The blood samples were then placed on ice and transferred back to the laboratory where the tubes were centrifuged for 25 min at approximately 1,800 *× g* and 4 °C. The plasma was then further aliquoted into individual microcentrifuge tubes to determine NEFA, β-HB, and cortisol concentration. Plasma NEFA concentration was determined using microtiter plates and a plate reader in a 2-reaction, enzyme-based assay (NEFA-HR(2), Fujifilm Wako Diagnostics, USA, Richmond, VA) as previously described by Johnson and Peters (1993). The internal control used in each plate was a commercially available control lyophilized human serum (Control Serum I, Fujifilm Wako Diagnostics, USA, Mountain View, CA). The inter-assay variation was 5.4%, and intra-assay variation was 4.8%. β-HB concentrations were quantified using the LiquiColor procedure (Beta-Hydroxybutyrate LiquiColor Test Endpoint, Stanbio Laboratory, Boerne, TX). Inter-assay variation was 6.0%, and intra-assay variation was 7.5%. Plasma cortisol concentration was quantified using a commercially available radioimmunoassay kit (MP Biomedicals, LLC., Solon, OH). The minimum level of detection was 10.0 ng/mL. The inter-assay variation was 10.6%, and intra-assay variation was 5.8%.

### Statistical Analysis

Calf body weight, average daily gain, NEFA concentration, β-HB concentration, cortisol concentration, and calf collar data (distance, walking time, non-walking time, AUI, and average speed) were all analyzed as a completely randomized design using the MIXED procedure of SAS (9.4, SAS Inst. Inc., Cary, NC). Calf was considered the experimental unit as the intranasal treatments were applied to each calf individually. The model included treatment, day, and their interaction as fixed effects, and calf ID within treatment as the random effect. A covariance structure was used to account for the error’s correlation due to the repeated measures over time. Different covariance structures were tested and the one with the lowest AIC was selected. For the variables with unequally spaced time points (body weight, NEFA, β-HB, and cortisol), a spatial power covariance structure was utilized. The compound symmetry covariance structure was used for average daily gain, distance walked, walking time, non-walking time, AUI, and average speed. The Kenward–Roger method was used to calculate the denominator degrees of freedom. Significant differences were declared when *P* ≤ 0.05, and tendencies were declared at 0.05 > *P* ≤ 0.10.

## RESULTS

### Global Positioning System Collar Data

The data obtained from the Garmin collars are presented in Table 2. There was a day effect for all collar parameters including distance walked (*P* < 0.01), walking time (*P* < 0.01), non-walking time (*P* < 0.01), AUI (*P* < 0.01), and average speed (*P* < 0.01). Distance walked was greatest in both treatment groups on the day of weaning and decreased on each subsequent sampling day (*P* < 0.01). Similarly, calves spent the most time walking on the day of weaning and decreased the time devoted to walking on each sampling day with their least amount of walking time on day 14 after weaning (*P* < 0.01). AUI was the greatest in both treatment groups on day 7 after weaning, followed by day 0, and exhibiting the lowest AUI on day 14 after weaning (*P* < 0.01). There was a day effect for average speed, such that walking speed increased with each sampling day in both treatment groups (*P* < 0.01). There was also a tendency for a treatment × day effect for average speed (*P* = 0.09), with calves in both treatments increasing their average speed with each subsequent sampling day and calves in the OXT treatment having a greater average speed on day 14 after weaning. There were no treatment effects for distance walked (*P* = 0.50), walking time (*P* = 0.66), non-walking time (*P* = 0.39), AUI (*P* = 0.51), or average speed (*P* = 0.51). Similarly, there were no treatment × day effects observed for distance walked (*P* = 0.82), walking time (*P* = 0.80), non-walking time (*P* = 0.88), or AUI (*P* = 0.84).

**Table 2.** Least squares means ± SEM for distance walked (km), time devoted to walking^†^ (h), AUI^‡^, and average walking speed (m/s) for groups of calves weaned with intranasal oxytocin (*n* = 10) or intranasal saline (*n* = 10) on days 0, 7, and 14 after weaning

	Oxytocin			Saline			*P*-Values		
	Day 0	Day 7	Day 14	Day 0	Day 7	Day 14	Trt	Day	Trt × Day
Distance	9.10 ± 0.38	2.69 ± 0.36	1.85 ± 0.36	9.58 ± 1.09	2.77 ± 0.38	1.90 ± 0.36	0.50	<0.01	0.82
Walking time	3.15 ± 0.13	0.86 ± 0.12	0.52 ± 0.12	3.19 ± 0.12	0.88 ± 0.13	0.55 ± 0.12	0.66	<0.01	0.80
Non-walking time	12.85 ± 0.13	15.14 ± 0.13	15.48 ± 0.13	12.81 ± 0.13	15.12 ± 0.13	15.45 ± 0.13	0.39	<0.01	0.88
AUI	0.63 ± 0.04	0.85 ± 0.04	0.34 ± 0.04	0.68 ± 0.04	0.87 ± 0.04	0.34 ± 0.04	0.51	<0.01	0.84
Average speed	0.82 ± 0.02	0.88 ± 0.01	0.96 ± 0.01	0.83 ± 0.01	0.91 ± 0.02	0.93 ± 0.01	0.51	<0.01	0.09

^†^Walking time, h = total time devoted to walking in 16 h.

^‡^AUI = total polygonal area of the weaning paddock utilized by the calf divided by the total area of the paddock.

### Performance and Plasma Metabolites

The body weight, average daily gain, plasma NEFA, β-HB, and cortisol concentration data are presented in Table 3. There were no treatment or treatment × day effects for body weight (*P* = 0.67; *P* = 0.82) or average daily gain (*P* = 0.70; *P* = 0.54); however, there was a day effect for these variables (*P* < 0.01). Both treatments did not differ (*P* = 0.52) in the amount of body weight lost from day 0 to day 1 and then gained weight on days 7 and 14. Calves did not differ in their average daily gains from day 0 to 7 (*P* = 0.73) and day 7 to 14 (*P* = 0.30), having greater gains in the second week after weaning. There was a treatment effect (*P* = 0.02) and a day effect (*P* < 0.01) for plasma NEFA concentration. Treatment groups did not differ (*P* = 0.81) in plasma NEFA concentration at the start of the study; however, the CON treatment had greater NEFA concentrations throughout the remainder of the study compared with the OXT treatment (*P* = 0.03). Additionally, both treatment groups had their least NEFA concentration on the day of weaning and increased their concentration on day 1. By day 14 of the study, NEFA concentrations in both treatment groups had not yet returned to baseline concentrations recorded on day 0. There was no treatment effect (*P* = 0.36) for plasma β-HB concentration; however, there was a day effect (*P* < 0.01) observed with both treatment groups increasing their β-HB concentrations from day 1 to 14. There was also a tendency for a treatment × day effect for β-HB (*P* = 0.10). Although calves in both treatments increased their β-HB concentration as the days after weaning increased, the CON treatment tended to have a greater increase compared with the OXT treatment. There were no treatment (*P* = 0.99) or treatment × day (*P* = 0.32) effects for cortisol; however, there was a day effect (*P* < 0.01) with both treatment groups having their greatest cortisol concentrations on day 1.

**Table 3.** Least squares means ± SEM for body weight in (kg), average daily gain^†,‡^ (kg/d, plasma NEFA (mmol/L), β-HB (μmol/L), and cortisol (ng/mL) concentration for groups of calves weaned with intranasal oxytocin (*n* = 10) or intranasal saline (*n* = 10) on days 0, 1, 7, and 14 after weaning

	Oxytocin				Saline				*P*-Values		
	Day 0	Day 1	Day 7	Day 14	Day 0	Day 1	Day 7	Day 14	Trt	Day	Trt × Day
Body Weight	194.4 ± 2.72	184.9 ± 2.72	197.2 ± 2.72	202.1 ± 2.72	193.0 ± 2.72	182.3 ± 2.72	196.7 ± 2.72	200.3 ± 2.72	0.67	<0.01	0.82
Average daily gain	—	—	0.40 ± 0.27^†^	1.54 ± 0.27^‡^	—	—	0.53 ± 0.27^†^	1.14 ± 0.27^‡^	0.70	<0.01	0.54
NEFA	0.25 ± 0.06	0.42 ± 0.06	0.45 ± 0.06	0.41 ± 0.06	0.24 ± 0.06	0.64 ± 0.06	0.57 ± 0.06	0.59 ± 0.05	0.02	<0.01	0.22
β-HB	183.1 ± 22.00	262.0 ± 22.00	305.1 ± 22.00	308.3 ± 22.00	153.2 ± 22.00	286.4 ± 22.00	316.5 ± 22.00	334.9 ± 22.00	0.36	<0.01	0.10
Cortisol	10.0 ± 0.68	12.7 ± 0.68	10.0 ± 0.68	10.0 ± 0.68	11.3 ± 0.68	11.4 ± 0.68	10.0 ± 0.68	10.0 ± 0.68	0.99	<0.01	0.32

^†^Average daily gain (kg/d) from day 0 to 7.

^‡^Average daily gain (kg/d) from day 7 to 14.

## DISCUSSION

Our results do not support the hypothesis that intranasal oxytocin administered on the day of weaning will decrease the distance walked and time devoted to walking by calves. It is possible that there were no effects observed in any of the collar data evaluated (distance, walking time, non-walking time, AUI, and average speed) because the two treatment groups were housed together in the same pasture and may have synchronized their behaviors to act as one group. The housing of all calves in one group is a limitation to this study, as it has been demonstrated that cattle are a gregarious species known to be affected by behaviors of their conspecifics, leading to the transmission of behavior patterns throughout the group (Nicol, 1995). When several individuals respond to other animals in the herd by imitating their behaviors, the entire group eventually behaves synchronously (Sumpter, 2010). Postural synchrony among cattle in which members of a herd lie down or stand up at the same time has been well documented in animals at pasture (Benham, 1982). It is feasible that calves in both the oxytocin and saline treatment groups naturally synchronized their behavior, and this could be a reason that the GPS collar data are similar among treatment groups.

The lack of effect of intranasal oxytocin treatment on distance walked may be because of the short half-life, 8 to 12 min, in the blood of cattle (Wagner et al., 2020a). Although the dose rate of oxytocin used in the current study (0.66 IU/kg BW) may not have been sufficient to induce behavioral and metabolic changes in the calves, the dose rate of oxytocin treatment was double the rate used in *Bos indicus* heifers exposed to restraint and isolation stress (0.3 IU oxytocin/Kg BW) and calves under transportation (Wagner et al., 2020b; Wagner et al., 2021). 

**Figure 1.**
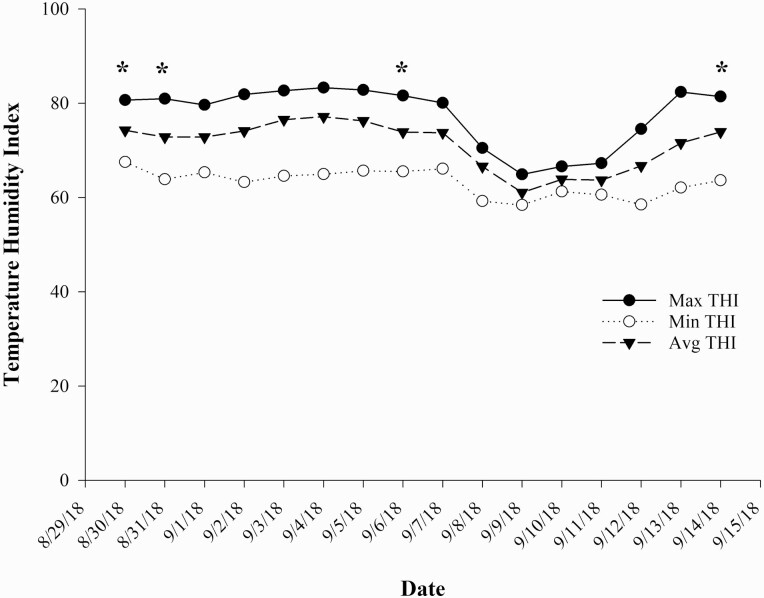
Minimum, maximum, and mean daily THI for the 15-d period of the weaning study. *Indicates day 0, and days 1, 7, and 14 after weaning.

Calves in the OXT and CON groups walked 9.12 and 9.58 ± 0.141 km on day 0, respectively. In pasture systems with adequate forage and temperate climates, cattle will typically walk 4 to 8 km each day (Squires, 1981; Nickles et al., 2020). In moderate and extreme climatic environments, cattle have been recorded to walk 7 to 14 km/d (Hodder and Low, 1978) and even up to 20 km/d (Squires, 1981) to find forage. Therefore, while the distances reported in the current study on day 0 are greater than previously reported for cattle in pasture-based systems, this is not surprising for abruptly weaned calves as walking behaviors are typically increased after cow–calf separation (Veissier and le Neindre, 1989; Price et al., 2003; Haley et al., 2005). Calves in the present study seemed to walk shorter distances on days 7 and 14 after weaning compared with previously reported distances walked by cattle in temperate climates. The THI data from the duration of the 2-wk study are shown in Fig. 1. *Bos taurus* cattle exhibit mild heat loads when the THI exceeds 72 (Du Preez et al., 1990; Armstrong, 1994) and exhibit severe head loads when the THI is around 79 (Hahn and Mader, 1997). The average maximum THI throughout the duration of the present study was 77.6, and the average minimum was 63.2. On the days that GPS collars were placed on calves (days 0, 7, and 14), maximum THI ranged from 75 to 81. Therefore, the mild to severe heat stress (THI greater than 72) during the study may have acted as an additional stressor combined with weaning stress and may explain why calves walked seemingly short distances on days 7 and 14 after weaning compared with previously reported walking distances on days 7 and 14 after weaning (Nickles et al., 2020).

The present study does not support the hypothesis that intranasal oxytocin administered at weaning would decrease NEFA concentrations. Both treatments had a similar mean plasma NEFA concentration on day 0, since blood samples were obtained immediately after separation from their dams. While both treatment groups had elevated plasma NEFA concentrations on day 1, the CON treatment had greater elevations compared with the OXT treatment, and this difference was present throughout the remainder of the study on days 7 and 14. The NEFA concentrations in the present study were slightly greater than that reported by Lynch et al. (2012) on the day of weaning. Lynch et al. (2012) demonstrated a similar increase in plasma NEFA concentration from day 0 to 2 after weaning; however, by day 7, the plasma NEFA concentration began to return to baseline concentrations. In the present study, both treatment groups had an increase in plasma NEFA concentration on day 1; however, the magnitude of increase was greater in the CON treatment compared with the OXT treatment. By day 14 after weaning, the OXT treatment began to exhibit decreased plasma NEFA concentration, whereas the CON treatment did not. Furthermore, cortisol and NEFA concentrations have been reported to be positively correlated, as cortisol helps to facilitate NEFA mobilization from adipose tissue during periods of stress to increase energy availability (Sapolsky et al., 2000). We speculate that the intranasal OXT may have initially suppressed the HPA axis and release of cortisol on the day of weaning which in turn could have prevented as great of a mobilization of fatty acids in the OXT-treated calves. We also observed similar plasma β-HB concentration as those reported by Lynch et al. (2012) on the day of weaning. Lynch et al. (2012) also demonstrated that plasma β-HB increased as the days after weaning increased with abruptly weaned calves in their study having greater β-HB concentration at day 14 after weaning compared with the day of weaning. As we observed a continued increase in plasma β-HB concentration as days after weaning increased, it is possible that calves were still adapting to weaning stress and a new diet and it may take longer than 14 d for calves to return to baseline concentration. To our knowledge, there has not been an established cut-point for determining ketosis for beef calves; however, in dairy cows, a β-HB concentration of ≥1,200 µmol/L is recognized as a risk factor for ketosis (Oetzel, 2004). This concentration of β-HB considered for risk of ketosis is still much greater than the concentration observed in the present study.

It is also possible that combined with the behavior synchronization of the calves, the sample size may have been too small to detect any treatment effects on walking distances, body weight, and β-HB. A power analysis was completed in SAS with the treatment means, standard error of the mean, sample size per group, and an alpha of 0.05 for walking distance, body weight, and plasma β-HB concentration. A power of 0.75, 0.53, and 0.65 was observed for walking distance, body weight, and plasma β-HB concentration, respectively. This indicates that 10 animals per treatment were not a great enough sample size to determine treatment differences for these variables. When a power analysis was completed for NEFA and cortisol, however, the observed power was 0.99 and 0.98, respectively. This is an indication that we did have sufficient power for both of these parameters.

In summary, calves given intranasal oxytocin at weaning did not experience any behavioral changes related to walking distances or time devoted to walking. It is possible that the behavioral synchrony previously observed in cattle may have a greater effect than the administration of oxytocin on walking behaviors. There was no evidence for a difference in body weight, average daily gain, and plasma β-HB and plasma cortisol concentrations; however, plasma NEFA concentration was greater for the CON treatment compared with the OXT treatment. Based on the findings of this study, further research is warranted to determine oxytocin’s mode of action in reducing plasma NEFA concentration and if the administration of oxytocin can induce significant changes in production.
